# Low energy electrodynamics of CrI_3_ layered ferromagnet

**DOI:** 10.1038/s41598-021-02918-4

**Published:** 2021-12-03

**Authors:** Luca Tomarchio, Salvatore Macis, Lorenzo Mosesso, Loi T. Nguyen, Antonio Grilli, Mariangela Cestelli Guidi, Robert J. Cava, Stefano Lupi

**Affiliations:** 1 Department of Physics, Sapienza University, Piazzale Aldo Moro 5, 00185 Rome, Italy; 2 INFN section of Rome, P.Le Aldo Moro, 2, 00185 Rome, Italy; 3 INFN - Laboratori Nazionali di Frascati, via Enrico Fermi 54, 00044 Frascati, Rome Italy; 4 Department of Chemistry, Princeton University, Princeton, NJ 08544 USA

**Keywords:** Electronic properties and materials, Magnetic properties and materials, Quantum fluids and solids

## Abstract

We report on the optical properties from terahertz (THz) to Near-Infrared (NIR) of the layered magnetic compound CrI_3_ at various temperatures, both in the paramagnetic and ferromagnetic phase. In the NIR spectral range, we observe an insulating electronic gap around 1.1 eV which strongly hardens with decreasing temperature. The blue shift observed represents a record in insulating materials and it is a fingerprint of a strong electron-phonon interaction. Moreover, a further gap hardening is observed below the Curie temperature, indicating the establishment of an effective interaction between electrons and magnetic degrees of freedom in the ferromagnetic phase. Similar interactions are confirmed by the disappearance of some phonon modes in the same phase, as expected from a spin-lattice interaction theory. Therefore, the optical properties of CrI_3_ reveal a complex interaction among electronic, phononic and magnetic degrees of freedom, opening many possibilities for its use in 2-Dimensional heterostructures.

## Introduction

Three-dimensional (3D) layered van der Waals (vdW) crystals^1–3^ are systems preserving the 2-Dimensional (2D) phenomenology while guaranteeing significant advantages over their applications in 3D bulk devices^4–6^. Their emerging functional properties are associated with non conventional electronic behaviors like excitonic interactions and dynamics^7^ and spin/valley physics^8,9^. Recently, these exotic electronic properties combined with intrinsic ferromagnetic order have been found in vdW transition metal halides like CrI_3_ and CrCl_3_^10–15^. Here, ferromagnetism may sustain novel phases of matter, like the Quantum anomalous Hall Effect (QAH)^16,17^ or the spin liquid state^18,19^, opening numerous opportunities for magneto-optical applications^20–22^. Moreover, bulk layered vdW magnets can be exploited as substrates, interfacial layers and tunnel barriers for engineering magnetic proximity effects^23,24^ and designing novel spintronic applications^25,26^.

**Figure 1:**
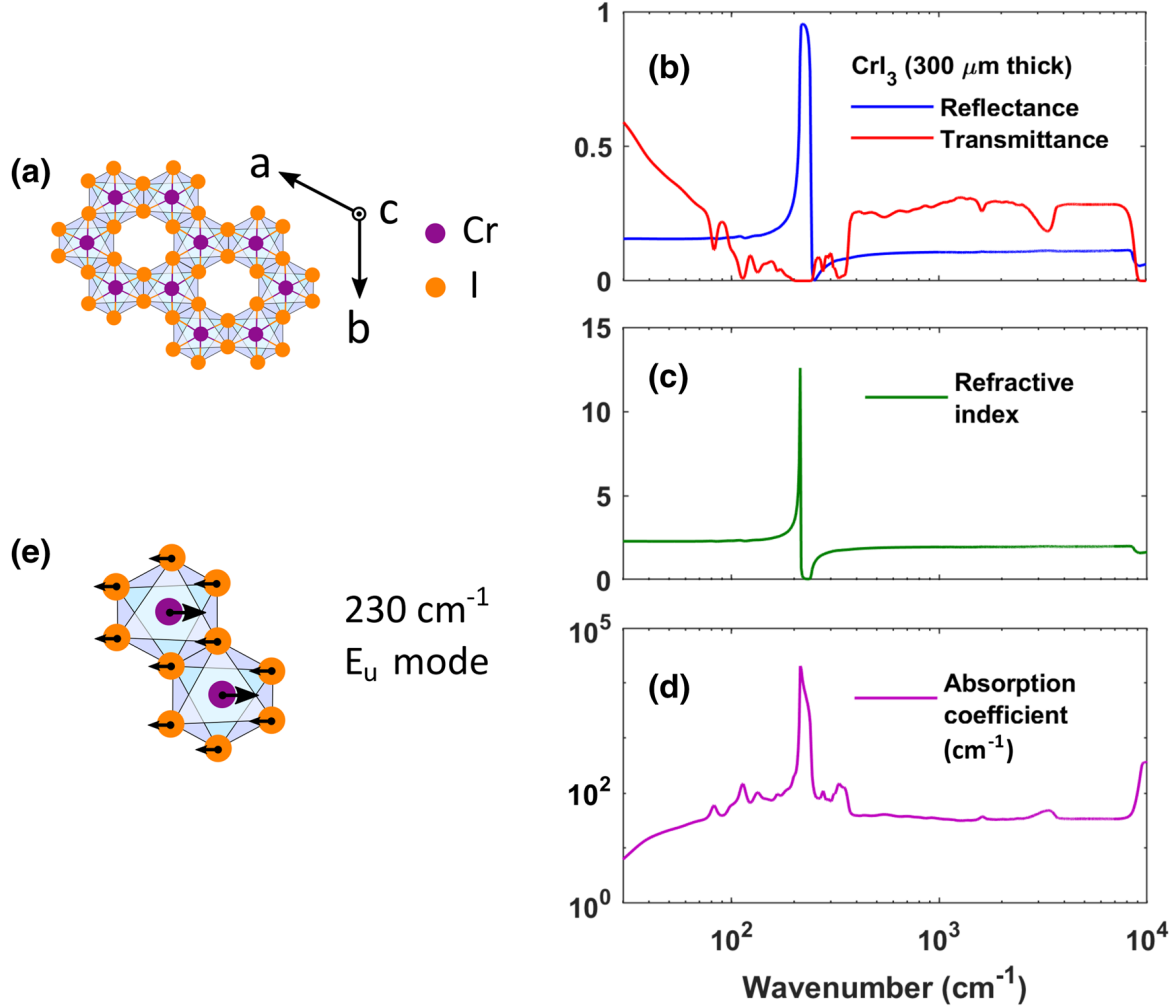
Infrared spectroscopy measurements of a CrI_3_ single crystal. (**a**) Top view of the crystal structure of CrI_3_. The Cr and I atoms are bonded to form honeycomb ordered layers. The arrows indicate the crystal axes. (**b**) Optical reflectance and transmittance of a 300 μm thick CrI_3_ crystal at 300 K. The reflectance is dominated by a single phonon mode at 230 cm^−1^. The measured transmittance highlights instead a plethora of far infrared vibrational modes and a band-gap around 9200 cm^−1^. (**c**) Real part of the refractive index of CrI_3_ at 300 K. (**d**) Absorption coefficient at 300 K of CrI_3_. (**e**) In-plane phonon mode of the Cr atoms, associated to the strong vibrational mode at 230 cm^−1^ in the bulk CrI_3_.

Chromium trihalide CrI_3_ has been shown to be a cleavable magnetic material with a great tunability of its magnetic properties with thickness^27–29^. Bulk CrI_3_ is a layered c-axis anisotropic ferromagnetic insulator with a Curie temperature of 61 K and a rhombohedral layer stacking below ~ 220 K, where a first order structural phase transition converts the unit cell from a monoclinic room temperature phase^30^. In each layer, the Cr atoms form a honeycomb structure (Fig. 1a), with each of them surrounded by six Iodine atoms in an octahedral coordination^27^. Remarkably, few-layer CrI_3_ has been proved to host anti-ferromagnetic order between the layers, with a Néel critical temperature of 45 K and a monoclinic stacking^28^.

CrI_3_ has been theoretically predicted^31,32^ to host strong interactions among electronic, phononic and magnetic degrees of freedom, including a strong spin-orbit coupling (SOC)^33–35^, with the appearance of exotic responses like nonreciprocal magneto-electric effects^26,36,37^. As a result, CrI_3_ is a candidate material to host subtle, low energy, emergent phases of matter^38,39^. Indeed, recent results^40–42^ have proved how an isostructural compound like α-RuCl_3_ is able to host low energy fractionalized excitations reminiscent of a Kitaev spin liquid phase. Moreover, neutron magnetic scattering measurements on CrI_3_^43^ have suggested the presence of topological magnon dispersions^44^, with the appearance of magnon edge states, analogous to topological insulators for electronic systems. Additional studies on the magnetic order revealed how the breaking of the spin-rotation invariance may be caused by large SOC, rather than the crystal field anisotropy^67^. All these results lead to a very complex picture of all degrees of freedom interactions in CrI_3_.

Although theoretical and experimental data suggest CrI_3_ to be a candidate material for hosting subtle emergent phases of matter, its bulk electronic and vibrational properties have never been investigated, at least in our knowledge. In this work we address this gap, by investigating the optical properties of a bulk CrI_3_ single crystal in its whole phase diagram covering both the paramagnetic and ferromagnetic phases. In particular, we determine the electrodynamic response of CrI_3_ from THz to near infrared (NIR), while tuning the temperature across the structural and magnetic phase transitions, down to the liquid helium temperature. The NIR response of CrI_3_ shows the presence of an optical gap associated to the crystal-field splitting of the Cr *d*-bands ($d_{xy,x_2-y_2}$ and $d_{xz,yz}$)^30,45^, which is subjected to a giant frequency blue shift (nearly 2000 cm^−1^), from 300 to 5 K. Although this giant hardening is mainly related to a strong electron-phonon interaction, a further blue shift is observed below the ferromagnetic temperature, also suggesting a strong coupling among electronic and magnetic degrees of freedom. In the far infrared, we show the presence of single and multiple-phonon excitations superimposed to a broad absorption background. We study the temperature dependence of these excitations and their modification with the appearance of a magnetic order.

## Results and discussion

CrI_3_ single crystals were synthesized by a chemical vapor transport technique (see Methods). The crystal structure of CrI_3_ is shown in Fig. 1a. The Chromium (Cr) and Iodine (I) atoms are bonded to form honeycomb ordered layers. The arrows indicate the *a*, *b* and *c* crystal axes. The bulk crystal structure of CrI_3_ at room temperature is described by a monoclinic (space group C2/m) unit cell. Below the structural phase transition at T$_{struc}$ $\sim 220$ K, this changes to a rhombohedral symmetry (space group R$\bar{3}$)^30^.

Reflectance (R) and Transmittance (T) measurements were performed in a broad spectral range from THz (20 cm^−1^) to NIR (15000 cm^−1^) (~ 2.5 meV–1.86 eV) and temperatures from 5 to 300 K. The spectroscopy set-up is discussed in the Methods section. In Fig. 1b we report the room temperature R and T of a CrI_3_ single crystal with a 300 μm thickness. Fig. 1c shows the real part of the refraction index, while Fig. 1d the corresponding absorption coefficient, both extracted through the RefFit Kramers-Kronig consistent fitting process^46^. The reflectance spectrum is dominated by a strong phonon absorption near 230 ^−1^, which can be associated to the in-plane E$_u$ collective oscillations of Cr atoms^31^ (see Fig. 1e). In the far-infrared transmittance, we are instead able to resolve additional low energy absorption peaks, extending to nearly 400 cm^−1^ which are related to multi-phonon excitations (see below). Above 400 cm^−1^, a flat transmittance (absorbance) is observed, extending up to the crystal-field electronic gap that can be observed both in transmittance and reflectance at room-T around 9200 cm^−1^ (1.14 eV). The transmittance minima (broad weak maxima in the absorption coefficient, Fig. 1d), appearing on the IR plateau at about 1600 and 3600 cm^−1^, are instead associated to the bending and stretching vibrations of few intercalated water molecules among the CrI_3_ layers^47^. Indeed, layered systems are common hosting materials for various intercalant species, ranging from small ions to atoms and molecules^48^.

### Temperature dependence of the electronic gap

**Figure 2:**
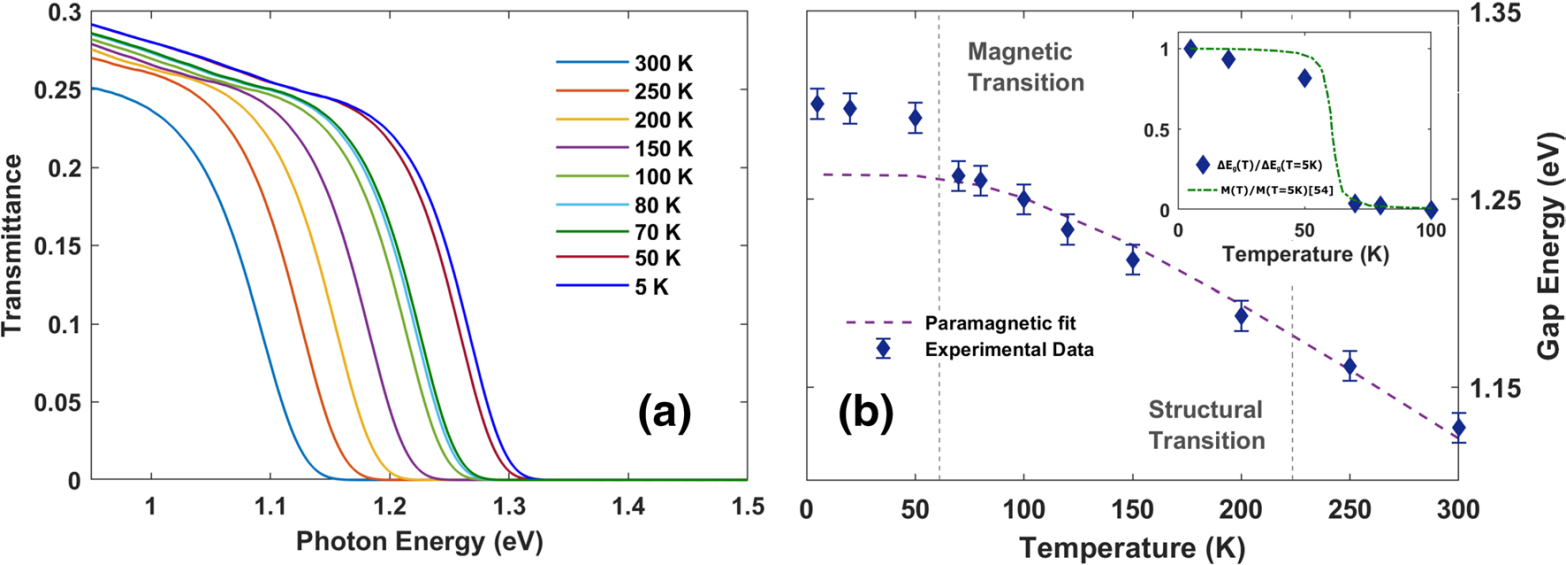
Variation of the electronic band gap of CrI_3_ with temperature. (**a**) Measured NIR transmittance for a 300 μm CrI_3_ slab at various temperatures. A strong red shift towards higher temperatures is clearly visible. (**b**) Optical band-gap as a function of temperature. The dotted line at 61 K separates the values above and below the Curie temperature, where a discontinuity in the band gap energy shift is highlighted. The paramagnetic phase was fitted through the model in Eq. (1), proving the presence of strong electron-phonon correlations in CrI_3_ (see Table 1). The inset shows the comparison of the extra-gap values $\Delta E_g(T)$ (normalized to $\Delta E_g(5\;K)$, see text) and the magnetization order parameter *M*(*T*) normalized at the lowest temperature^54^.

**Figure 3:**
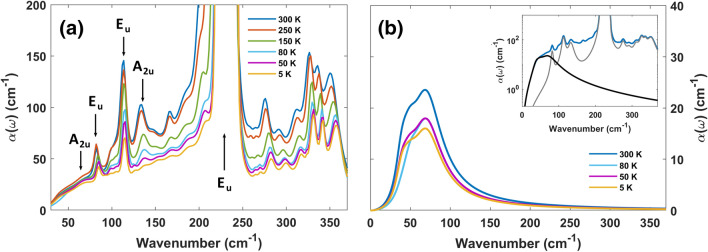
Temperature dependence of the far infrared vibrational modes of CrI_3_. (**a**) Absorption coefficient at various temperatures as extracted from the transmittance measurements fitting process. A general transparency is observed with decreasing temperatures, along with the disappearance of three modes at very low temperatures. The arrows highlight the main vibrational modes predicted in accordance with the $D_{3d}$ point group symmetry. (**b**) Absorption spectrum after removing the predicted in-plane phonon resonances and the few major peaks lacking a clear identification, as computed by the best fitting process of the transmittance. A general absorptive background is highlighted across the low energy spectrum, showing an increasing transparency with the lowering temperature. Visible differences in the absorption background behavior can be highlighted while crossing the Curie temperature. The inset shows the contribution of the absorptive background (black curve) to the total absorption coefficient at 300 K (blue curve). The gray curve shows the contributions coming from the known phonon peaks.

The temperature dependent transmittance measurements in the NIR spectral region are highlighted in Fig. 2a. Here, a huge blue shift (nearly 2000 cm^−1^) of the electronic gap E$_g$ can be observed with decreasing temperature from 300 K to 5 K. E$_g$(T) values are extracted by a linear fitting of the decreasing transmittance through its intercept with the frequency axis^49^. E$_g$(T) as a function of temperature is reported in Fig. 2b. In this Figure, both the ferromagnetic Curie temperature T$_{c}$ and the structural transition temperature T$_{struc}$ have been indicated by vertical dotted lines. While across the structural transition the electronic gap presents a smooth behavior, at the paramagnetic/ferromagnetic transition a discontinuity appears with a robust increase in the gap value below $T_c$. Both the lattice expansion and the electron-phonon interaction may induce a temperature dependence of the electronic gap^50,51^. Both terms can be modeled through the Manoogian and Leclerc empirical equation^50,52^

1 $$\begin{aligned} E_g(T) = E_g(T=0) + UT^s-V\epsilon \left( \coth (\epsilon /2k_BT)-1\right) \end{aligned}$$

where *U*, *s*, *V* and $\epsilon$ are temperature independent coefficients. *U* and *V* are the coupling constants weighting the lattice expansion and electron-phonon interaction contributions, respectively, while $\epsilon$ is an energy averaging all the acoustic and optical phonons. E$_g$ data in Fig. 2b for the paramagnetic phase have been fitted through Eq. (1). The result is shown in Fig. 2b through a dashed purple line. Fitting coefficients in Eq. (1) are presented in Table 1, compared to other semiconductors from literature. The lattice expansion, parametrized by *U*, has been found to give a negligible contribution to the temperature dependence of E$_g$. The strongest effect is thus given by the electron-phonon interaction, whose intensity is measured by the coefficient *V*, higher than the one found in most of the known semiconductors (see Table 1). The further blue shift of the electronic band gap below the Curie temperature suggests a further dependence of the electronic gap from the magnetic degrees of freedom. In order to quantify this discontinuity, we define an extra gap-value $\Delta E_g(T)$ as the difference between the actual gap value $E_g(T)$ and that corresponding to the paramagnetic extrapolation below $T_c$, $\Delta E_g(T) = E_g(T)-E_{fit}(T)$. $E_{fit}(T)$ is determined by using the Eq. (1) fitting process (see Fig. 2b). At 5 K (the minimum temperature we reach in our optical measurements), $\Delta E_g(T)=35 \; {\text{meV}}$. This value cannot be related to a modification of the electron-phonon interaction, since the phonon spectrum is unaltered across the transition (see Fig. 3a). From the theoretical point of view, a recent work^31^ calculates the electronic structure of CrI_3_ monolayers both in the magnetic and non-magnetic phase. This suggests that the electronic band structure is strongly perturbed by the magnetic state and depends on the magnetization (*M*) easy axis direction. In particular, the electronic gap is larger when *M* is along the c-axis than in the ab plane of the CrI_3_ structure. This result, calculated for CrI_3_ monolayer, seems to be valid also for bulk CrI_3_^31^. In this framework, magnetic measurements^54^ show that magnetization in bulk single crystals develops along the c-axis. In order to establish a correlation between optical and magnetic data, in the inset of Fig. 2b we show $\Delta E_g(T)$ normalized to $\Delta E_g(T=5 \;K)$. In the same inset, we also plot the magnetic moment along the c-axis^54^ normalized to its lowest temperature (5 K), *M*(*T*)/*M*(5 *K*). Both quantities follow a very similar trend, suggesting that the extra gap value is related to the development of the magnetic state. These results highlight a complex degrees of freedom interplay in CrI_3_, suggesting that the electronic gap hardening might be related to a non-trivial coupling between the electrons and the magnetic order^26,29,35^.

**Table 1:** Coefficients for the band gap frequency shift of semiconductors as a function of temperature, as obtained by the model of Eq. (1).

	$E_g(0)$ (eV)	$\epsilon$ (meV)	V
CrI_3_ (paramagnetic)	1.26	26	4.68
CuGaS_2_^53^	2.5	38	1.53
CdGeP_2_^52^	1.89	–	3.2
CdGeAs_2_^52^	0.595	–	1.27
ZnSnSb_2_^52^	0.66	–	3.74
Ge^51^	0.74	–	2.77
Si^51^	1.17	–	2.74
GaAs^51^	1.52	–	3.14

The symbol “–” highlights missing values from literature. The resulting temperature dependence for CrI_3_ is shown in Fig. 2.

### Far infrared response

The far-IR absorption coefficients at different temperatures are shown in Fig. 3a in an expanded vertical scale. The spectra are composed by several peaks located between 70 and 360 cm^−1^ and we observe an overall decrease of the absorption by reducing T. Due to the van der Waals nature of the CrI_3_ crystal and the in-plane polarization of the incident radiation in this experiment, a single layer model for the lattice vibrations is expected to describe the experimental phonon absorption peaks. Indeed, CrI_3_ layers can be described by the $D_{3d}$ point group symmetry^31,55^, which predicts five IR-allowed transitions, namely three $E_u$ modes and two $A_{2u}$ modes, three inactive modes (one $A_{1u}$ and two $A_{2g}$), and six Raman-active modes (two $A_{1g}$ and four $E_g$). Raman spectra have already been measured in previous works^15,56–61^, revealing the presence of magnons and a plethora of magneto-optical effect. The corresponding Raman peaks at room-T are reported in Table 2, together with numerical calculations (at 0 K)^61–63^ and the IR absorption peaks observed at room-T in our experiment, as measured by absorption peak maxima. In the theoretical calculations, the heavier iodine atoms are predicted to dominate the phonon spectrum below 150 cm^−1^^31,64^, therefore being related to the strong absorption peaks at 82 cm^−1^, 114 cm^−1^ ($E_u$ modes) and 133 cm^−1^ ($A_{2u}$ mode). At higher energies, above the strong absorption at 230 cm^−1^ ($E_u$ symmetry, mainly due to Cr vibrations), a series of peaks can be seen in Fig. 3a, with a strong spectral weight from 300 to 360 cm^−1^. These higher frequency excitations are not predicted by the ab-initio calculations for CrI_3_^31,62,64^. However, their frequencies can be captured by a linear combination of Raman and IR fundamental modes as reported in Table 2, suggesting an important role of anharmonicity in the phonon spectrum of CrI_3_.

Further differences from the $D_{3d}$ point group symmetry predictions can be found in the presence of extra absorption shoulders at nearly 100, 150, 170 cm^−1^ and near the strong E$_u$ peak at 230 cm^−1^. The presence of these excitations has been investigated in recent DFT calculations of monolayer CrI_3_^64^, showing their dependence from the magnetic ordering. Indeed, their temperature dependence (they nearly disappear below T$_c$) is not trivial. A similar result is obtained for the $A_{2u}$ predicted in-plane phonon at 133 cm^−1^ (as measured at $T=300\;{\text{K}}$), which seems to disappear at low temperatures. These results have been explained in terms of a strong spin-phonon coupling^64^, which predicts the appearance of a gap in the phonon density of states between the two E$_u$ modes at 113 and 230 cm^−1^.

**Table 2:** CrI_3_ vibrational modes frequencies (in cm^−1^) at 300 K.

Raman-active	IR-active	IR-active (two-phonons)
52 [50.1] ($E_{g}$)	60 [56.8] ($A_{2u}$)	276 ($230E_u+52E_g$)
79 [76.1] ($A_{1g}$)	82 [80.3] ($E_u$)	291 ($230E_g+60A_{2u}$)
99 [101.8] ($E_{g}$)	113 [114.3] ($E_u$)	310 ($230E_u+79A_{1g}$)
105 [107.5] ($E_g$)	133 [133.3] ($A_{2u}$)	326 ($230E_u+99E_g$)
128 [129] ($A_{1g}$)	230 [225.3] ($E_u$)	337 ($230E_u+105E_g$)
230 [241.1] ($E_g$)		347 ($230E_g+113E_u$)

The values in the quadratic brackets highlight the in-plane Raman- and IR-active modes at 0 K, as obtained by DFT results^31^. The first column shows the experimental Raman modes^61,63^. The IR-active experimental modes obtained in this work are shown in the second and third columns.

The low-energy (THz) side of the absorption coefficient suggests the presence of a broad background. Its general shape and temperature dependence can be obtained by a best fitting process of the absorption coefficient at various temperatures, taking into account the phonon peaks previously discussed (see the inset of Fig. 3b for an example of fitting at 300 K). An absorption background has been observed in the THz range in $\alpha$-RuCl_3_^65,66^. Although strongly debated, this background has been mainly associated to Kitaev spin liquid excitations. In CrI_3_, at variance with $\alpha$-RuCl_3_, this broad absorption, centered around 70 cm^−1^, is already present at room-T and decreases with reducing T, nearly saturating below T$_c$ (see Fig. 3a). The broad temperature-dependent THz background could have electronic, lattice, or magnetic origins. Bulk CrI_3_ is a very good electric insulator with an electronic gap around 1.2 eV. This implies that we do not expect thermally-induced free electrons in the material (in particular at low-*T*), which can affect the absorption in the THz range. This excludes an electronic origin of the THz background. In RuCl_3_ Kitaev-like material, where many theoretical calculations exist for the 3D magnetism, a similar background (of magnetic origin) increases with decreasing *T*. In CrI_3_, instead, it is a decreasing function of *T*, nearly reaching an intensity saturation at $T_c$. Moreover, it is located around 10 meV, an energy larger than the exchange magnetic energy in CrI_3_ ($J \sim 3 \;meV$)^67^. This difference, associated to the decreasing *T*-dependence, suggests a non-magnetic origin. The last mechanism, i.e., acoustic phonon assisted absorption, has been proposed some years ago to explain extra absorptions in the THz and sub-THz regions in alkali-halides^68,69^. The extra absorption corresponds to processes in which optical modes are excited by photons concomitantly with the absorption of acoustic modes at high wavevectors. Due to the quasi-continuum distribution of acoustic modes, one expects a broad absorption band, which depends on *T* due to the modes *T*-dependence. In conclusion, the characteristic background energy (nearly 10 meV) and its temperature dependence seem to rule out both a magnetic and electronic origins, suggesting instead an acoustic assisted mechanism at the main contributor.

## Conclusions

In this work we have investigated the optical response of a CrI_3_ single crystal from Terahertz to Near-Infrared at various temperatures, both in the paramagnetic and ferromagnetic phase. We have observed an insulating optical gap around 1.1 eV at 300 K which strongly depends on temperature, showing a robust hardening for decreasing T. This hardening is due to a huge electron-phonon interaction which is reinforced below the Curie critical temperature at nearly 60 K. This indicates a complex interaction scenario among lattice, electronic and magnetic degrees of freedom in CrI_3_ system.

By studying the far-IR/THz absorption spectrum we have observed several phonon peaks that have been assigned in agreement to the $D_{3d}$ point group symmetry and DFT calculations. Our finding of some magnetic-sensitive peaks could be the first experimental evidence that these lowest-frequency absorptive terms exhibit strong spin-phonon coupling. The phonons absorption is also superimposed to a broad background already visible at 300 K and having a decreasing magnitude with *T*. This is at variance with the isostructural $\alpha$-RuCl_3_ compound, where the absorption background increases at low-*T* and has been associated mainly to Kitaev spin liquid excitations. Although CrI_3_ has been suggested to be a candidate to host similar fractionalizated excitations, as indicated by recent theoretical results^70^ and by the discovery of gapped Dirac magnon dispersions^43^, this absorption background could have a different origin probably related to the strong lattice anharmonicities. Although we studied the optical properties of CrI_3_ in its bulk form, their dependence on the magnetic transition suggests that also for few-layer CrI_3_ the electronic excitations should be strongly correlated to the magnetic ones. This suggests a complex interplay among different degrees of freedom in CrI_3_ that, when controlled, could induce a rich variety of quantum phenomena. In conclusion, the present experiment clarifies the low-energy electrodynamics of bulk CrI_3_, fixing a solid point for the investigation of its optical behavior in the dimensionality crossover from 3D to 2D.

## Methods

### Sample Growth

CrI_3_ single crystals were synthesized by a chemical vapor transport technique. A 1 g mixture of the stoichiometric ratio of Cr metal and I_2_ pieces (Alfa Aesar, 99.99%) was packed in a sealed evacuated quartz glass tube (22 cm long and 16 mm wide) and heated in a three zone furnace, set at zone temperatures 650, 550, and 600 °C, for one week. The “charge” was placed in the 650 °C zone. Many CrI_3_ crystals were formed in the 550 °C zone. The crystals are stable in air for a few hours.

### Optical characterization

Optical measurements at various temperatures have been performed through a Bruker Vertex 70v Infrared interferometer, coupled with different detectors and beamsplitters covering the spectral region from THz (20 cm^−1^) to NIR (15000 cm^−1^). A liquid He-cooled bolometer has been used for measurements from 20 up to 600 cm^−1^, while a room-temperature pyroelectric detector has been used for the higher frequencies. The optical measurements have been taken at various temperatures through a He-cooled ARS cryostat.

## References

[1] Wang QH, Kalantar-Zadeh K, Kis A, Coleman JN, Strano MS (2012). Electronics and optoelectronics of two-dimensional transition metal dichalcogenides. Nat. Nanotechnol..

[2] Manzeli S, Ovchinnikov D, Pasquier D, Yazyev OV, Kis A (2017). 2D transition metal dichalcogenides. Nat. Rev. Mater..

[3] Reedijk, J. & Poeppelmeier, K *From Elements to Applications* (Elsevier, Comprehensive Inorganic Chemistry II, 2013).

[4] Liu CW, Östling M, Hannon JB (2014). New materials for post-Si computing. MRS Bull..

[5] Lemme MC, Li L-J, Palacios T, Schwierz F (2014). Two-dimensional materials for electronic applications. MRS Bull..

[6] Radisavljevic B, Radenovic A, Brivio J, Giacometti V, Kis A (2011). Single-layer MoS_2_ transistors. Nat. Nanotechnol..

[7] Mak KF, Shan J (2016). Photonics and optoelectronics of 2D semiconductor transition metal dichalcogenides. Nat. Photon..

[8] Felser C, Fecher GH, Balke B (2007). Spintronics: a challenge for materials science and solid-state chemistry. Angewandte Chemie.

[9] Schaibley JR (2016). Valleytronics in 2D materials. Nat. Rev. Mater..

[10] Dillon JF, Kamimura H, Remeika JP (1966). Magneto-optical properties of ferromagnetic chromium trihalides. J. Phys. Chem. Solid.

[11] Wang, H., Eyert, V. & Schwingenschlögl, U. Electronic structure and magnetic ordering of the semiconducting chromium trihalides CrCl_3_, CrBr_3_, and CrI_3_. *J. Phys. Condens. Matter* **23**, 116003 (2011) 10.1088/0953-8984/23/11/116003 21358033

[12] Niu B (2020). Coexistence of Magnetic Orders in Two-Dimensional Magnet CrI_3_. Nano Lett..

[13] Pollini I (1998). Electron correlations and hybridization in chromium compounds. Solid State Commun..

[14] Bermudez VM, McClure DS (1979). Spectroscopic studies of the two-dimensional magnetic insulators chromium trichloride and chromium tribromide-I. J. Phys. Chem. Solids.

[15] Jin W (2020). Observation of the polaronic character of excitons in a two-dimensional semiconducting magnet CrI_3_. Nat. Commun..

[16] Tokura Y, Yasuda K, Tsukazaki A (2019). Magnetic topological insulators. Nat. Rev. Phys..

[17] Chang C-Z (2013). Experimental observation of the quantum anomalous hall effect in a magnetic topological insulator. Science.

[18] Jackeli G, Khaliullin G (2009). Mott Insulators in the strong spin-orbit coupling limit: from Heisenberg to a quantum compass and Kitaev models. Phys. Rev. Lett..

[19] Knolle J, Kovrizhin DL, Chalker JT, Moessner R (2014). Dynamics of a two-dimensional quantum spin liquid: signatures of emergent majorana fermions and fluxes. Phys. Rev. Lett..

[20] Pershan PS (1967). Magneto-optical effects. J. Appl. Phys..

[21] Freiser M (1968). A survey of magnetooptic effects. IEEE Trans. Magn..

[22] Haider T (2017). A review of magneto-optic effects and its application. Int. J. Electromag. Appl..

[23] Zhao W (2020). Magnetic proximity and nonreciprocal current switching in a monolayer WTe_2_ helical edge. Nat. Mater..

[24] Zhong D (2017). Van der Waals engineering of ferromagnetic semiconductor heterostructures for spin and valleytronics. Sci. Adv..

[25] Seyler KL (2018). Ligand-field helical luminescence in a 2D ferromagnetic insulator. Nat. Phys..

[26] Liu, Z. et al. Observation of nonreciprocal magnetophonon effect in nonencapsulated few-layered CrI_3_. *Sci. Adv.* **6**, 43, eabc7628 (2020) 10.1126/sciadv.abc7628 PMC7608833 33097544

[27] Liu Y (2019). Thickness-dependent magnetic order in CrI_3_ single crystals. Sci. Rep..

[28] Huang B (2017). Layer-dependent ferromagnetism in a van der Waals crystal down to the monolayer limit. Nature.

[29] Gudelli VK, Guo G-Y (2019). Magnetism and magneto-optical effects in bulk and few-layer CrI_3_: a theoretical GGA + U study. New J. Phys..

[30] McGuire MA, Dixit H, Cooper VR, Sales BC (2015). Coupling of crystal structure and magnetism in the layered, ferromagnetic insulator CrI_3_. Chem. Mater..

[31] Webster L, Liang L, Yan J-A (2018). Distinct spin-lattice and spin-phonon interactions in monolayer magnetic CrI_3_. Phys. Chem. Chem. Phys..

[32] Zhang Y (2019). Switchable magnetic bulk photovoltaic effect in the two-dimensional magnet CrI_3_. Nat. Commun..

[33] Bacaksiz C, Šabani D, Menezes RM, Milošević MV (2021). Distinctive magnetic properties of CrI_3_ and CrBr_3_ monolayers caused by spin-orbit coupling. Phys. Rev. B.

[34] Chen L (2020). Magnetic anisotropy in ferromagnetic CrI_3_. Phys. Rev. B.

[35] Stavropoulos PP, Liu X, Kee H-Y (2021). Magnetic anisotropy in spin-3/2 with heavy ligand in honeycomb Mott insulators: application to CrI_3_. Phys. Rev. Res..

[36] Sun Z (2019). Giant nonreciprocal second-harmonic generation from antiferromagnetic bilayer CrI_3_. Nature.

[37] Pervishko AA (2020). Localized surface electromagnetic waves in CrI_3_-based magnetophotonic structures. Opt. Express.

[38] Tokura Y, Kawasaki M, Nagaosa N (2017). Emergent functions of quantum materials. Nat. Phys..

[39] Keimer B, Moore JE (2017). The physics of quantum materials. Nat. Phys..

[40] Nasu J, Knolle J, Kovrizhin DL, Motome Y, Moessner R (2016). Fermionic response from fractionalization in an insulating two-dimensional magnet. Nat. Phys..

[41] Sandilands LJ, Tian Y, Plumb KW, Kim Y-J, Burch KS (2015). Scattering Continuum and Possible Fractionalized Excitations in α-RuCl_3_. Phys. Rev. Lett..

[42] Wang Z (2017). Magnetic Excitations and Continuum of a Possibly Field-Induced Quantum Spin Liquid in α-RuCl_3_. Phys. Rev. Lett..

[43] Chen L (2018). Topological spin excitations in honeycomb ferromagnet CrI_3_. Phys. Rev. X.

[44] Costa, A. T., Santos, D. L. R., Peres, N. M. R. & Fernandez-Rossier, J. Topological magnons in CrI_3_ monolayers: an itinerant fermion description. *2D Mater.* **7**, 045031 (2020)

[45] Wu Z, Yu J, Yuan S (2019). Strain-tunable magnetic and electronic properties of monolayer CrI_3_. Phys. Chem. Chem. Phys..

[46] Kuzmenko AB (2005). Kramers-Kronig constrained variational analysis of optical data. Rev. Sci. Instrum..

[47] Freda M (2005). Transmittance fourier transform infrared spectra of liquid water in the whole mid-infrared region: temperature dependence and structural analysis. Appl. Spectrosc..

[48] Jung Y, Zhou Y, Cha JJ (2016). Intercalation in two-dimensional transition metal chalcogenides. Inorg. Chem. Front..

[49] Ghobadi N (2013). Band gap determination using absorption spectrum fitting procedure. Int. Nano Lett..

[50] Manoogian A, Woolley JC (1984). Temperature dependence of the energy gap in semiconductors. Can. J. Phys..

[51] Van Zeghbroeck BV (2010). Principles of Semiconductor Devices and Heterojunctions.

[52] Bhosale J (2012). Temperature dependence of band gaps in semiconductors: electron-phonon interaction. Phys. Rev. B.

[53] Levcenco S (2010). Temperature dependence of the exciton gap in monocrystalline CuGaS_2_. Physica B Condens. Matter.

[54] Liu Y, Petrovic C (2018). Three-dimensional magnetic critical behavior in CrI_3_. Phys. Rev. B.

[55] Bermudez VM (1976). Unit-cell vibrational spectra of chromium trichoride and chromium tribromide. Solid State Commun..

[56] Jin W (2018). Raman fingerprint of two terahertz spin wave branches in a two-dimensional honeycomb Ising ferromagnet. Nat. Commun..

[57] Jin W (2020). Tunable layered-magnetism-assisted magneto-Raman effect in a two-dimensional magnet CrI_3_. PNAS.

[58] Cenker J (2021). Direct observation of two-dimensional magnons in atomically thin CrI_3_. Nat. Phys..

[59] Huang B (2020). Tuning inelastic light scattering via symmetry control in the two-dimensional magnet CrI_3_. Nat. Nanotechnol..

[60] Guo X (2021). Structural monoclinicity and its coupling to layered magnetism in few-layer CrI_3_. ACS Nano.

[61] McCreary A (2020). Distinct magneto-Raman signatures of spin-flip phase transitions in CrI_3_. Nat. Commun..

[62] Larson DT, Kaxiras E (2018). Raman spectrum of CrI_3_: an ab initio study. Phys. Rev. B.

[63] Ubrig, N. et al. Low-temperature monoclinic layer stacking in atomically thin CrI_3_ crystals. *2D Mater. ***7**, 015007 (2020)

[64] Wang, K. *et al.**Magnetic Order-dependent Phonon Properties in 2D Magnet CrI*_3_ (Nanoscale, Advance Article, 2021). 10.1039/d1nr00820j 34125128

[65] Little A (2017). Antiferromagnetic resonance and terahertz continuum in α-RuCl_3_. Phys. Rev. Lett..

[66] Reschke S (2017). Electronic and phonon excitations in α-RuCl_3_. Phys. Rev. B.

[67] Chen L (2020). Magnetic anisotropy in ferromagnetic CrI_3_. Phys. Rev. B.

[68] Stolen R, Dransfeld K (1965). Far-infrared lattice absorption in alkali halide crystals. Phys. Rev..

[69] Sparks M, King DF, Mills DL (1982). Simple theory of microwave absorption in alkali halides. Phys. Rev. B.

[70] Xu, C., Feng, J., Xiang, H. & Bellaiche, L. Interplay between Kitaev interaction and single ion anisotropy in ferromagnetic CrI_3_ and CrGeTe_3_ monolayers. *npj Comput. Materi.* **4**, 57 (2018)

